# Automated crack identification in structures using acoustic waveforms and deep learning

**DOI:** 10.1186/s43065-024-00102-2

**Published:** 2024-08-11

**Authors:** Mohamed Barbosh, Liangfu Ge, Ayan Sadhu

**Affiliations:** Department of Civil and Environmental Engineering, The Western Academy for Advanced Research, Western University, London, ON N6A 3K7 Canada

**Keywords:** AE, Deep learning, Prediction of damage severity, Localization of damage, Concrete elements

## Abstract

Structural elements undergo multiple levels of damage at various locations due to environments and critical loading conditions. The level of damage and its location can be predicted using acoustic emission (AE) waveforms that are captured from the generation of inherent microcracks. Existing AE methods are reliant on the feature selection of the captured waveforms and may be subjective in nature. To automate this process, this paper proposes a deep-learning model to predict the damage severity and its expected location using AE waveforms. The model is based on a densely connected convolutional neural network (CNN) that offers superior feature extraction and minimal training data requirements. Time-domain AE waveforms are used as inputs of the proposed model to automate the process of predicting the severity of damage and identifying the expected location of the damage in structural elements. The proposed approach is validated using AE data collected from a concrete beam and a wooden beam and plate. The results show the capability of the proposed method for predicting the level of damage with an accuracy range of 92-95% and identifying the approximate location of damage with 90-100% accuracy. Thus, the proposed method serves as a robust technique for damage severity prediction and localization in civil structures.

## Introduction

Structural elements such as columns, beams, and slabs, comprised of different structural materials, are subjected to varying levels of damage during their operational time due to unexpected structural or environmental loads. In order to avoid any catastrophic failure, structural health monitoring (SHM) techniques can be applied to localize and detect the severity of damage in structures, assisting the engineers and owners with safety enhancement and timely maintenance for structures. Acoustic emission (AE) is one of the powerful SHM methods that can identify damage in various structural materials due to its sensitivity to microcracks and its capability to track the initiation and propagation of cracks. AEs are elastic waves generated by the rapid release of energy that can be measured using an array of sensors mounted on the monitored system subjected to active damage. Typical AE parameters such as amplitude, duration, signal strength, etc., can be extracted and utilized to detect the damage in the inspected material [35]. However, these AE parameters may provide inaccurate or misleading information about the damage location and its severity due to the influence of background noise in the measured AE data. Therefore, special attention needs to be taken to the raw AE signal features in different structural materials to automate the process of damage identification. This study proposes a method to identify the approximate location and predict the severity of damage using a deep learning method augmented with the time series response of AE waveforms.

AE is a passive nondestructive testing approach [14, 15, 43] that has been applied to monitor various engineering systems and materials, such as mechanical systems [2, 10, 19], and [20] and structural materials [1, 7, 27, 30, 40], and [42]. For example, Carnì et al. [11] proposed the AE analysis-based technique to monitor the crack initiation and propagation in concrete elements subjected to static loads. The relationship between stress response and typical AE parameters was investigated using the applied method and used as a damage indicator. Burud and Chandra Kishen 6 proposed an AE-based method to detect damage in concrete elements. The AE data collected from concrete beams were analyzed using the proposed method, and the b-value of the measured AE signal was extracted and then utilized to detect the fault pattern. In another study, Christensen et al. [12] applied AE methodology and digital image correlation techniques to predict the internal crack and track the propagation of the crack in concrete slabs. Experimental studies were undertaken to evaluate AE activity and digital image correlation from concrete slabs under loading testing. The results showed the capability of the combined method to detect and track damage propagation in concrete elements.

Singh et al. [36] utilized AE parameters to predict the initiation and propagation of the damage in the reinforced concrete beam-column joints subjected to cyclic loading. This study investigated the relationship between some typical AE parameters and the initiation and propagation of cracks and predicted the type of crack in concrete beam-column joints. The authors concluded that standard AE parameters can be considered to obtain some indicators about damage occurrence,however, they cannot identify the location of damage due to the change in wave velocity of AE waveforms. In another study, Tonelli et al. [39] studied the application of the AE technique to monitor a real-life concrete bridge under different loading conditions. A real-life concrete bridge in Italy was monitored using an AE system where AE signals were analyzed to detect and classify damages in the monitored bridge. It was concluded that the AE parameters analyses can provide helpful information on the damage present, predict damage level, and type in concrete elements. The authors mentioned that future works could be conducted on experimental specimens to verify the performance of the applied AE technique.

Machorro-Lopez et al. [25] proposed a new method based on AE waveforms to evaluate the existing condition of concrete beams under flexural loading tests. The proposed method employed continuous wavelet transforms on AE waveforms to determine the wavelet energy, which can be used to evaluate the health condition of the concrete beam. The performance of the proposed method was validated using an experimental test to collect AE data from concrete beams. The authors concluded that the performance of the proposed method needs to be investigated using different structural elements and materials subjected to various damage scenarios. On the other hand, Noorsuhada [26] conducted a comprehensive review of AE methodologies that were applied to monitor damage in reinforced concrete structures subjected to fatigue. This study provided a detailed discussion on the application of AE parameters-based analyses that have been employed to detect and quantify fatigue damage in various concrete elements. The authors stated that the application of AE techniques to localize the damage in concrete structures is limited and needs to be investigated in the future.

The location of a crack in the monitored system subjected to different levels of damage can be predicted by localizing the source of AE events. For example, Zhang et al. [44] applied a new damage identification method based on AE data measured from a concrete beam. This method was applied to identify the location of the crack using the probability density field of AE data collected from a concrete beam. The results showed that the new method can predict the crack pattern and localize the damage with less error than conventional localization methods. The authors suggested that future work can be conducted on the applied method, considering different types of sensor layouts and investigating the relationship between the probability density of AE events and the crack width. In another study, Ma et al. [23] developed a method using AE data to monitor the concrete bridge and identify the internal cracks during the bearing replacement process. Traditional AE parameters such as counts and amplitude were analyzed to identify the initiation and propagation of cracks in the bridge during the bearing replacement, where analyzing the AE parameters was found to be able to capture the initiation and propagation of the damage in concrete structures.

Recently, there has been an increase in the use of wood products as structural elements which brings high attention to the researchers to study the behaviour and durability of timber elements. Hu et al. [17] studied the vibrational characteristics of different types of wood elements under various environmental and loading conditions using nondestructive testing. Four popular wood species were experimentally tested using hammer impact to investigate the effect of the wood types on fundamental frequency, dynamic modulus of elasticity, and static modulus of elasticity. The results showed a significant effect of the wood species on vibrational characteristics. In another paper, Hu and Zhang [18] used an AE-parameters-based method to study the failure process of a wood element subjected to tension load. An experimental test on southern yellow pine wood element was conducted to investigate the effect of crack tip location on some AE parameters (i.e., amplitude, counts and energy). The results showed that the initiation and propagation of cracks in the wood elements can be detected using the AE technique.

Barbosh et al. [7] applied a feature selection method that integrates the decomposition of AE signal by empirical mode decomposition method and extracting damage indicator by Shannon Entropy to predict the approximate location of different fault scenarios in both wooden and concrete elements using AE waveforms. AE waveforms collected from experimental tests on wooden elements and full-scale concrete elements were utilized to verify the performance of the applied method as a damage detection and localization tool. In another study, Nasir et al. [28] provided a critical review of the application of the AE monitoring system in the field of wood and timber elements. The authors explained the concept, the performance, and the real-life application of the AE method that is used to monitor wood and timber structures. Some challenges of the AE application were discussed, and some directions were provided for future studies in wooden structures. Also, the study explained the existing methods used to analyze AE data and stated the gaps in the current AE analysis methods. The authors recommended that further studies be conducted to monitor the existing condition of large-scale timber structures using advanced signal-processing and artificial intelligence techniques. However, most of the above-mentioned studies have considered either traditional AE parameters or feature extraction methods to predict and localize the damage in concrete elements, which can provide inaccurate damage localization due to the effect of noise in the measured data and time-consuming due to vast measured data and multiple manual processes.

Artificial Intelligence is a more powerful tool in data analysis than traditional techniques due to its capabilities of processing vast amounts of data, identifying intricate patterns, and making accurate predictions. For example, Das et al. [13] analyzed AE parameters to classify the type of cracks (e.g., shear and tensile modes) in concrete structures using a Support Vector Machine (SVM) where some typical parameters such as RA values and Average frequency are used as input. Shimamoto et al. [37] applied k-means clustering and random forest algorithms to evaluate the importance of some AE parameters to characterize the fracture behaviour of concrete elements where the authors concluded that among the AE parameters examined, rise time and centroid frequency emerged as the most pivotal for gaining insights into compressive fracture processes in concrete structures. Jierula et al. [21] used SVM to classify the source location of AE signals in three sensor groups. The experimental test was conducted on an in-service reinforced concrete column. Biswas et al. [8] combined wavelet transform and Random Forest methods to detect the damage location and predict the type of damage in a steel frame structure using AE data.

Besides traditional machine learning approaches, Zhang et al. [45] proposed a new method using time-frequency features of AE signals as input into the deep learning methods to classify the type of damage in concrete structures. The results showed that the *ResNet18* provided higher accuracy over the other lightweight convolutional neural networks (CNNs) such as *GoogleNet*, *EfficientNet-b0*, and *MobileNetV2*. Lately, Barbosh et al. [9] proposed a new approach to predict the presence and location of damage in structural elements by applying a CNN model to time-frequency images of AE waveforms measured from wooden and concrete structures. On the other hand, Mahajan and Banerjee 24 developed an AE source localization approach using an artificial neural network to identify the AE source zone in rail structures. AE signals measured from pencil lead break tests and actual damage in a rail were used to train and test the models. The results indicated that these deep-learning approaches are beneficial in real-time AE-based rail inspection. However, most of the aforementioned studies employed either a traditional machine learning approach for feature selection that might be inaccurate and subjective or 2D training data, which is time-consuming to collect and annotate. There have been limited studies that used deep learning approaches on the time series response of AE waveforms to localize and predict the level of damage in wood and concrete structures.

DenseNets have attracted great attention in the field of audio processing and classification [31, 38], and [5] and bridge health monitoring [33], and Alfaz et al. 3 in recent years owing to their exceptional ability to extract features, even under conditions with limited samples. For instance, Li et al. [22] suggested a DenseNet-based fully convolutional network (FCN) for automatic pixel-level detection for various types of damage on concrete structure surfaces. Similarly, using images captured from concrete structures, Qiao et al. [33] introduced an instance segmentation algorithm for surface damage detection that combines DenseNet with an expectation-maximization attention unit (EMAU). In addition to applications in computer vision, a recent study by Wang et al. [41] proposed an approach termed as SDI-DenseNet for structural damage detection by training on acceleration responses from the free decay of a frame structure. The above studies demonstrated the potential of DenseNet-like networks for learning deep features in images and basic time series. However, for more complex time series like AE waveforms collected from concrete and wooden structures, the effectiveness of densely connected CNN architectures in damage detection has seldom been reported and warrants further investigation. Moreover, most previous studies considered methods based on feature extraction or the traditional parameters of AE signal to identify the damage, which may require some manual process and involve time-consuming and computation-intensive analysis. Regarding the gaps recognized from the literature review, this paper proposes AE waveform-based method to predict the damage severity and potential location in concrete and wooden structures using an automated 1D adaptation of the densely connected CNN model, which is named AE-DenseNet without involving any manual process and multi-step analysis. Systematic experiments were conducted to verify the superiority of the proposed model in localizing and predicting the level of damage using the time series response of AE waveforms and without considering any typical AE parameters and feature extraction.

The paper is outlined as follows. The proposed methodology is presented first. Then, experimental studies using concrete and wooden elements are conducted to show the performance of the proposed method, and finally, the conclusions are drawn.

## Methodology

This section gives a background of *AE-DenseNet*, which is a specially adapted network based on the concept of DenseNets. The input of AE-DenseNet is the time-domain waveform signals, while the output is a one-hot vector indicating damage severity and location. Compared to traditional 1D CNN methods, *DenseNet* has more efficient feature extraction and reusing, which facilitates the learning of complex patterns over time [41]. In the following subsections, the theoretical background and advantages of DenseNet are briefly described first, and then the design of AE-DenseNet and its application to damage detection are introduced.

### DenseNet

CNNs have been widely used in signal processing due to their outstanding capabilities of feature extraction. Such capabilities increase with the number of convolutional layers. However, as CNNs become increasingly deep, the input information may vanish during forward propagation, while the gradients may vanish or explode during backward propagation. Therefore, it is unreliable to extract features from complex signals only by increasing the depth of a standard CNN.

**Fig. 1:**
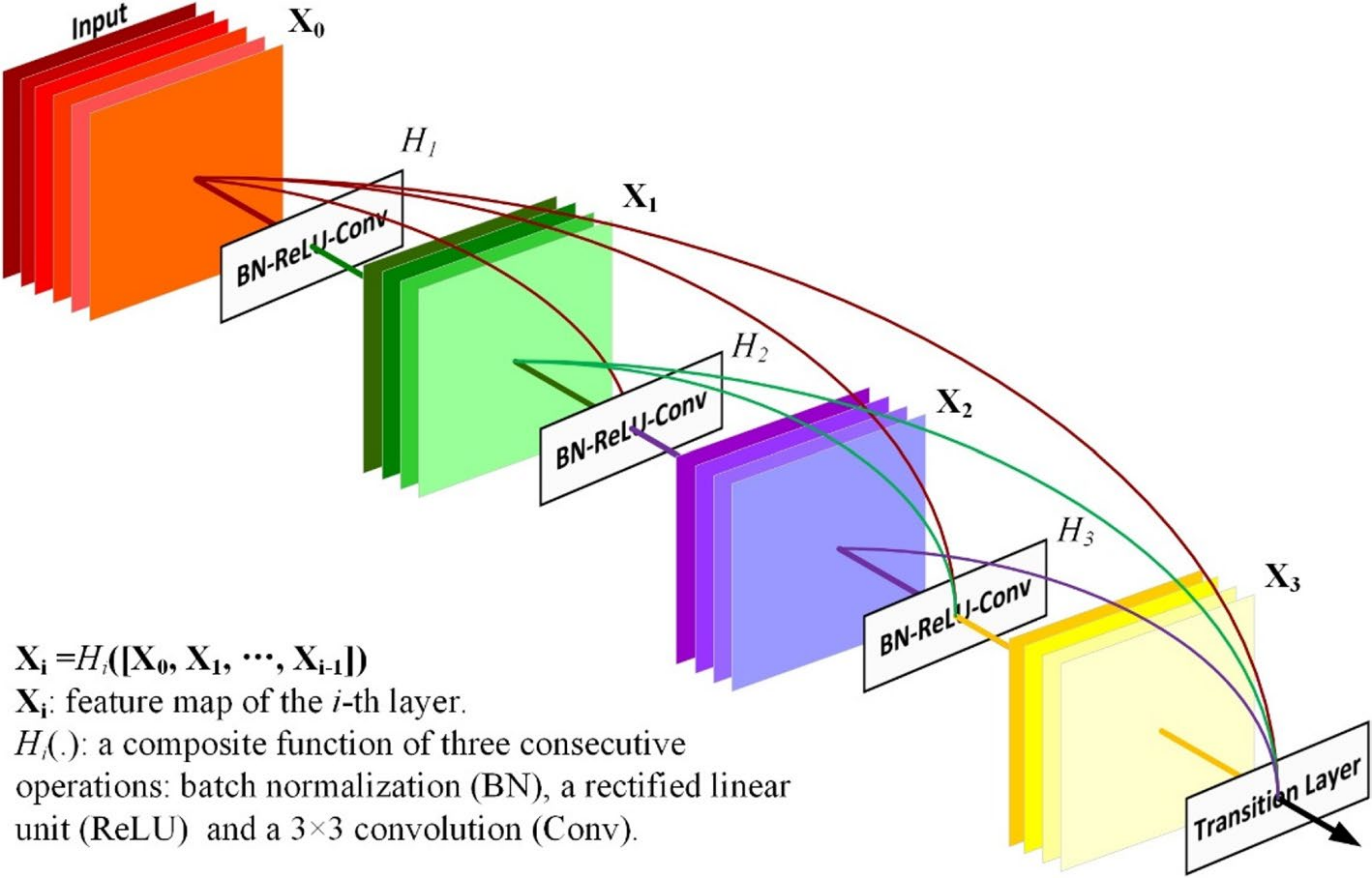
A 4-layer dense block with a growth rate of *k*=4

Although some architectures, such as Residual Networks (ResNets) [16] and Highway Networks [34], can solve the gradient vanishing/exploding problems by using skip connections, they require many more parameters than DenseNets. As shown in Fig. 1, to maximize the information flow between different layers, DenseNets connect all layers directly with each other. Each layer acquires supplementary feature maps from previous layers and forwards its feature map to all subsequent layers. In this way, *L* (*L+1*)/2 connections are introduced in an *L*-layer network instead of *L* in traditional CNN architectures. Generally, the number of feature maps produced through the function  *H*_*i*_ is defined as growth rate *k*. In the dense block, all the feature maps are the same size and concatenated without down-sampling. The down-samplings are achieved by transition layers with pooling between dense blocks. In addition, to improve computational efficiency and model compactness, a compression factor $\theta$ is introduced in DenseNets. $0\;<\theta\leq1$, indicating the percentage of feature maps to be reduced.

### Proposed method: AE-DenseNet

**Fig. 2:**
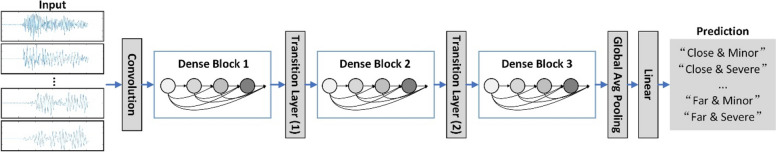
The architecture of AE-DenseNet with three dense blocks

As shown in Fig. 2, the proposed AE-DenseNet has three dense blocks that are connected with two transition layers. Different from the original DenseNets, which were specially designed for image classification, the inputs of AE-DenseNet are time-domain waveform signals collected from different AE sensors. Therefore, the proposed AE-DenseNet is designed with less dense blocks, improving its applicability to limited training data. All the waveform signals are of the same length related to the settings in the AE acquisition system. The proposed AE-DenseNet is an end-to-end damage detection model and has no requirement for conducting complex signal preprocessing on the input signals. As for the outputs, the waveform signals are classified into various groups representing damage scenarios with different damage severity and locations. The damage severity is labeled as "Minor" or "Severe" according to the number of hits detected by AE sensors, while damage locations are annotated as "Damage Far" and "Damage Close" using the relative distance from the sensor.

The specific architecture of the proposed AE-DenseNet is given in Table 1. The input signals are vectors in the size of (1, 5120), corresponding to the number of sample points per waveform. These vectors are first passed through a convolutional layer featuring a large filter to extract the preliminary signal patterns. In the forward propagation process, the feature maps are down-sampled by 25% every time passing through the transition layer, while the filter channels are compressed to reduce the number of model parameters by introducing the compression factor $\theta$. According to previous experience, $\theta = 0.5$ is suggested to achieve a trade-off between parameter saving and performance. To avoid underfitting, the number of layers of Dense Blocks (denoted as *L*) is optimized by a random search strategy [4] and set to 6 for the classification of damage location and damage severity. At the end of the network, a global average pooling layer is placed after the last Dense Block, followed by a fully connected softmax classifier. As all the training data is labeled with one-hot encoding, the categorical cross-entropy is chosen as the loss function.

**Table 1:** The architecture of the proposed network for damage classification

**Layers**	**Settings**	**Output Size**
Input	--	$1 \times 1\times 5120$
Convolution	$1\times 25 Conv, strides=(1, 4)$	$24 \times 1\times 1280$
Dense Block (1)	$$\left[\begin{array}{c}1\times 1 Conv\\ 1\times 15 Conv\end{array}\right]\times 6$$	$96 \times 1\times 1280$
Transition Layer (1)	$$\begin{array}{c}1\times 1 Conv\\ 1\times 4 Average Pooling, strides=(1, 4)\end{array}$$	$$\begin{array}{c}48 \times 1\times 1280\\ 48 \times 1 \times 320\end{array}$$
Dense Block (2)	$$\left[\begin{array}{c}1\times 1 Conv\\ 1\times 15 Conv\end{array}\right]\times 6$$	$120\times 1\times 320$
Transition Layer (2)	$$\begin{array}{c}1\times 1 Conv\\ 1\times 4 Average Pooling, strides=(1, 4)\end{array}$$	$$\begin{array}{c}60\times 1\times 320\\ 60\times 1\times 80\end{array}$$
Dense Block (3)	$$\left[\begin{array}{c}1\times 1 Conv\\ 1\times 15 Conv\end{array}\right]\times 6$$	$132\times 1\times 80$
Classification Layer	$$\begin{array}{c}Global Average Pooling\\ 1 \times class number fully connected, softmax\end{array}$$	$$class number$$

**Fig. 3:**
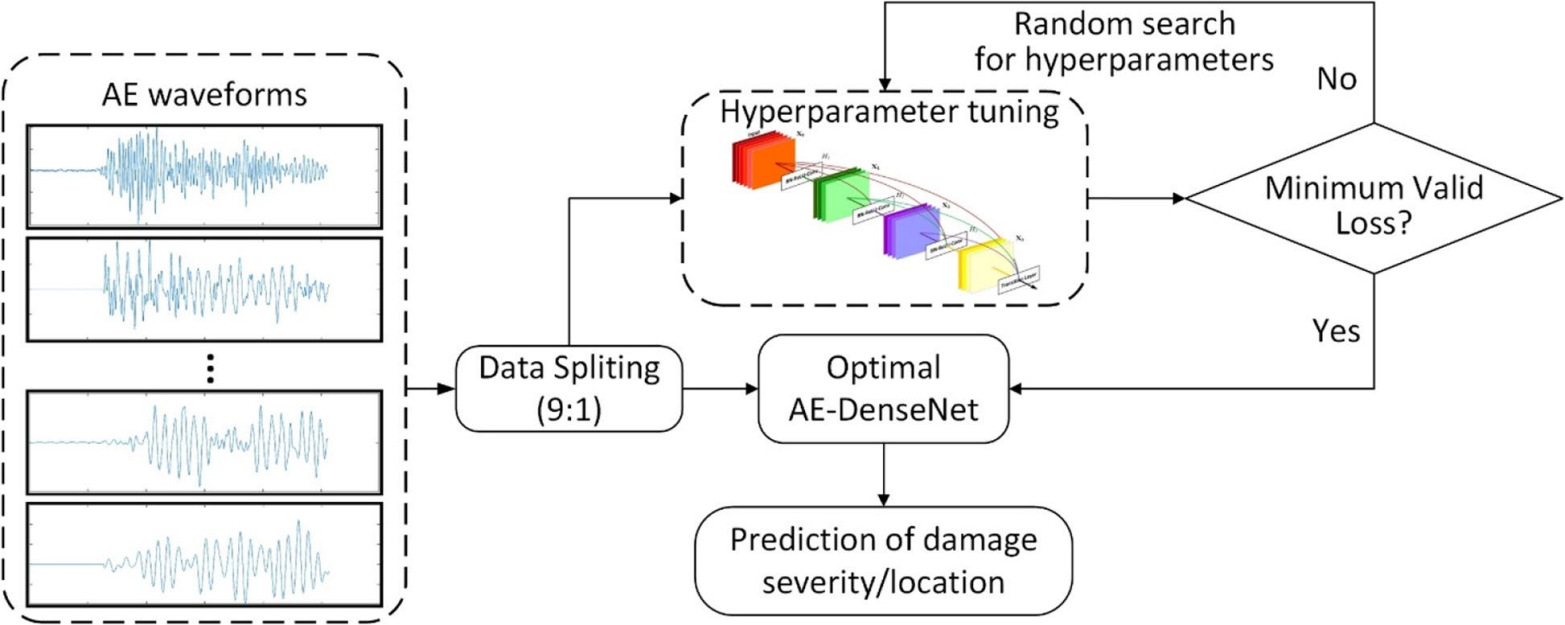
Flowchart of the proposed damage detection framework

The proposed AE-DenseNet employs the idea of dense connectivity from DenseNet that makes full use of training data. In particular, the fusion of feature maps between different layers greatly improves the utilization of information flow in the training. As all the fusions are essentially achieved through skip connections, the proposed approach can learn both low-level and high-level features of the input signals during training and has no requirement for signal preprocessing. Given this, AE-DenseNet is suitable for automated damage detection using complex acoustic signals and limited datasets collected from large-scale structures. Fig. 3 presents the flowchart of the proposed method.

### Metrics for performance evaluation

Once the AE-DenseNet has been trained, metrics for the evaluation of model performance can be extracted from the confusion matrix. In this study, the precision and recall rates were used to assess the trained network. Precision, also known as positive predictive value, indicates the ability of the model to accurately identify positive examples and to minimize false positives, as shown below [29]:

1 $$Precision=\frac{TP}{TP+FP}$$

where *TP* and *FP* are the true positive value and false positive value, respectively. Recall, also known as a true positive rate, measures the ability of the proposed network to find all the positive examples. It is defined as [29]:

2 $$Recall=\frac{TP}{TP+FN}$$

where *FN* is the false negative value.

## Experimental study using large-scale concrete beam

In order to comprehensively demonstrate the effectiveness of the proposed approach, this study investigated two challenging applications, specifically focusing damage detection on a large-scale reinforced concrete beam and a wooden board. This section provides details of the former experiment and an in-depth analysis of the implementation of AE-DenseNet.

### Experimental setup

**Fig. 4:**
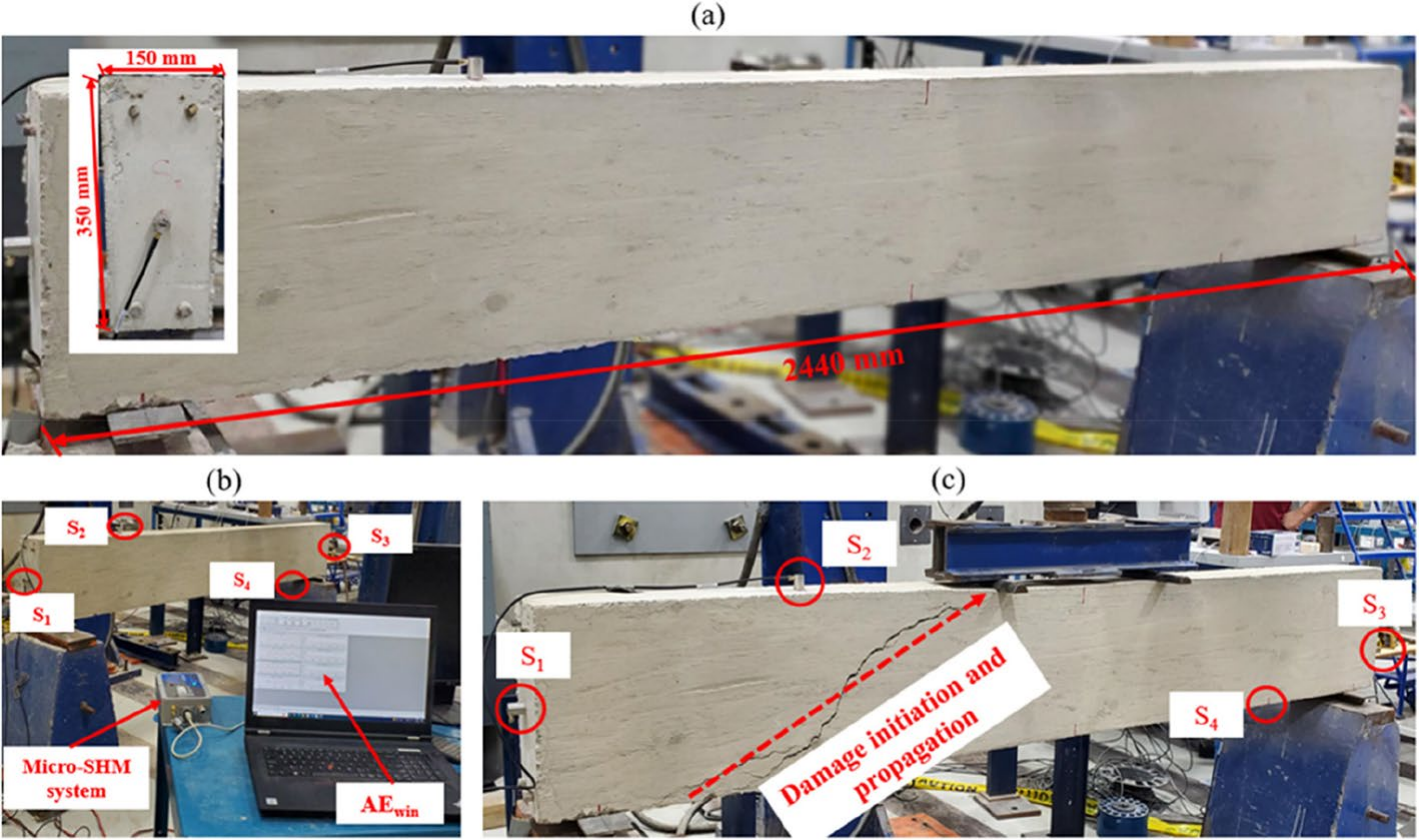
**a** Dimension of the beam, **b** AE monitoring system, and **c** Sensors placed on the beam and location of actual damage on the beam

In order to show the performance of the proposed method to identify the zonal location and predict the damage severity, an experimental test was conducted to collect AE data from a large reinforced concrete (RC) beam. As illustrated in Fig. 4(a), the beam possesses dimensions of 2440 mm in length, 150 mm in width, and 350 mm in thickness. The structural design mandates shear-induced failure, with reinforcement bars strategically placed solely on the upper and lower facets of the beam. It is cast using a formulated concrete mixture incorporating Portland cement, fine aggregate, and coarse aggregate. Following this, the curing process involves enveloping the beam with wet burlap for 28 days, culminating in a 28-day compressive strength of the concrete beam reaching 40 MPa. Fig. 4(b) illustrates the AE monitoring system deployed during the experimental test to capture AE activity. This system employs four AE sensors, denoted as S_1_, S_2_, S_3_, and S_4_. These four MISTRAS PK6I have a 55 kHz resonant frequency, a 35–65 kHz operating frequency range, and a peak sensitivity of 106 dB, which can operate in a temperature range between -35 to 80°C Physical Acoustics Corporation (PAC) [32]. The four PK6I sensors are connected to a Micro-SHM data acquisition system (DAQ) with four AE channels with a frequency bandwidth of 5 kHz-1 MHz and a sampling rate of 10 Msamples/s using amplification of 26 dB. This configuration was selected due to its capability to capture high-frequency AE data originating from micro-cracks in concrete. The Micro-SHM DAQ is linked to a computer unit for the purpose of transferring the acquired AE data. This process is conducted by utilizing AEwin software, which is configured with a threshold set at 40 dB. An MTS machine is employed to impose a three-point flexural load on the beam at a loading rate of 3.5 mm/min, continuing until damage initiates and propagates throughout the beam. The initiation and propagation of damage are closely monitored throughout the test. Visual observations reveal minor damage occurring at the test's outset, followed by the widening propagation of damage, indicating increased severity after a certain duration, as depicted in Fig. 4(c). Upon completion of the test and subsequent damage to the beam, the AE time series is acquired and stored on the computer for further analysis.

### Data Preparation

**Fig. 5:**
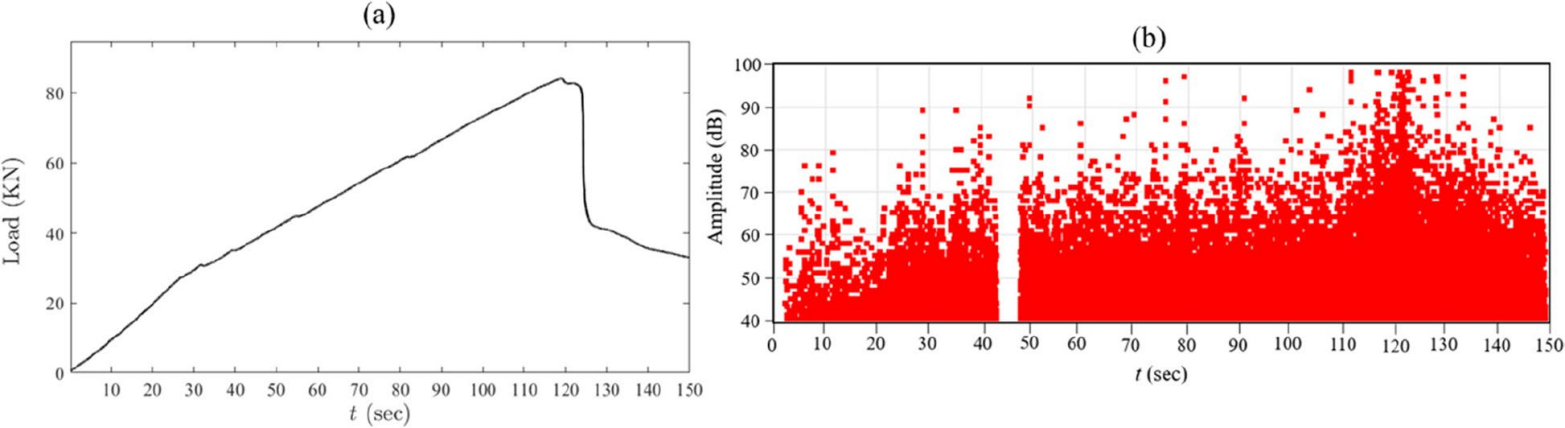
**a** Applied load curve and **b** AE activities collected from the four sensors placed on the beam during the loading process

For the concrete beam, the AE system continuously collected AE waveforms generated within the concrete beam throughout the whole servo loading process, yielding a total of 1365 signal segments captured by four AE sensors. Given the limited amount of measured data, the proposed AE-DenseNet is evaluated through two classification tasks: identifying the approximate location and predicting the severity of the damage. In order to identify the expected location of the damage on the beam referring to the location of the sensor mounted on the beam, the distance from the sensors to the damage is considered as the reference for damage localization where AE waveforms collected from S_1_ and S_2_ are labeled as "Damage Close" since the actual damage initiated and propagated at a distance ranges from 20 to 90 cm from S_1_ and S_2_, while those from S_3_ and S_4_ are labeled as "Damage Far" since the actual damage initiated and propagated at a distance ranges from 120 to 200 cm from S_3_ and S_4_. Regarding damage severity, the AE waveforms are categorized as either "Minor" or "Severe" where this classification is determined through a combination of visual monitoring conducted during the test and an assessment of the density and amplitude of the measured AE hits. The load-displacement curve of the beam is illustrated in Fig. 5(a). Notably, minor sudden changes were observed at the outset of the test, and a more pronounced alteration in continuity was evident at a specific point on the curve. These variations signify the initiation of damage (minor damage at the beginning of the test) and the subsequent propagation and expansion of damage (severe damage), respectively. Fig. 5 (b) shows AE activities measured from four AE sensors placed on the beam during the loading process. In Fig. 5 (b), there is an interval between 40-50 seconds where AE hits are absent, and this gap is a result of a temporary pause in the loading process during the test. Observably, the density and amplitude of AE activities are initially low at the test's outset, while the AE activities noticeably elevate at a certain time of the loading process (e.g., between 100-150 sec). This increase is attributed to the heightened level of damage, visually monitored throughout the test. Finally, the time series responses of these AE activities are acquired and labelled as severe damage for the last 50 sec, and the rest of the AE waveforms are labeled as minor damage for damage localization and severity prediction process where the amount of AE waveforms are for both categories (i.e., minor and severe) is shown in Table 2.

**Table 2:** Sample quantities of the collected dataset

Categories	Damage Close		Damage Far	
	Minor	Severe	Minor	Severe
Quantities	412	531	247	175

Since only limited experimental data is available for training the proposed AE-DenseNet, data augmentation was conducted in this study as well. To preserve the damaged features as much as possible, a strategy of random flipping was employed. The collected dataset was divided into the training set and validation set in a ratio of 9:1, and 50% of the training set was flipped for data augmentation. The sample quantities of the collected dataset for all the categories are listed in Table 2.

### Model Training and hyper-parameter Optimization

Two distinct models are separately trained for the classification tasks of damage severity and damage location to enable a comprehensive discussion of the learning capability of the proposed network for different features. The models are trained for 150 epochs on a computer equipped with an RTX 3060 GPU. To improve the accuracy and efficiency of the model, it is necessary to optimize the hyperparameters, including batch size, learning rate, and the layer number of the dense block, for the model architecture and training configurations. The key step of hyper-parameter optimization is to choose a good parameter combination ${\varvec{\lambda}}=\left\{{\lambda }^{\left(1\right)},{\lambda }^{\left(2\right)},...,{\lambda }^{\left(s\right)}\right\}$ from a designated search space $\Lambda$. *s* represents the number of hyperparameters. $\mathcal{L}$ is the categorical cross-entropy function that is used to evaluate deep learning models on the validation set ${X}^{\left(valid\right)}$. Then, the optimal combination ${{\varvec{\lambda}}}^{*}$ can be expressed as

3 $$\lambda\ast\;\approx\;\underset{\lambda\in\Lambda}{arg\;\min}\;\underset{x\in X^{\left(valid\right)}}{mean}\;\mathcal{L}\;\left(x;\;A_\lambda\left(X^{\left(train\right)}\right)\right)$$

where ${A}_{\lambda }$ represents the candidate model with the parameter combination $\lambda$, ${X}^{\left(train\right)}$ is the dataset used for training ${A}_{\lambda }$, and *x* is a sample selected from the validation set.

To search the hyperparameters more efficiently, the random search strategy [4], which is both reasonably efficient and simple, is applied in this study. The steps of the random search algorithm are as follows.

1. Define the range and distributions of hyperparameters. The ranges of learning rate, batch size and layer number are set as {0.0004, 0.0005, …,0.0015,0.0016}, {4,6,8,10,12,14} and {6,8,10,12}, respectively.
2. Determine the number of iterations in a random search. The iteration number is set as 200 in this study. In each iteration, a combination of hyperparameters is randomly selected from the defined search space.
3. Train the proposed model using the selected parameter set and evaluate its performance with the function $\mathcal{L}$.
4. Repeat steps 2 and 3 until the predetermined iteration number is reached. Iterate until the budget and finally obtain the best combination ${{\varvec{\lambda}}}^{*}$ for the model.

**Fig. 6:**
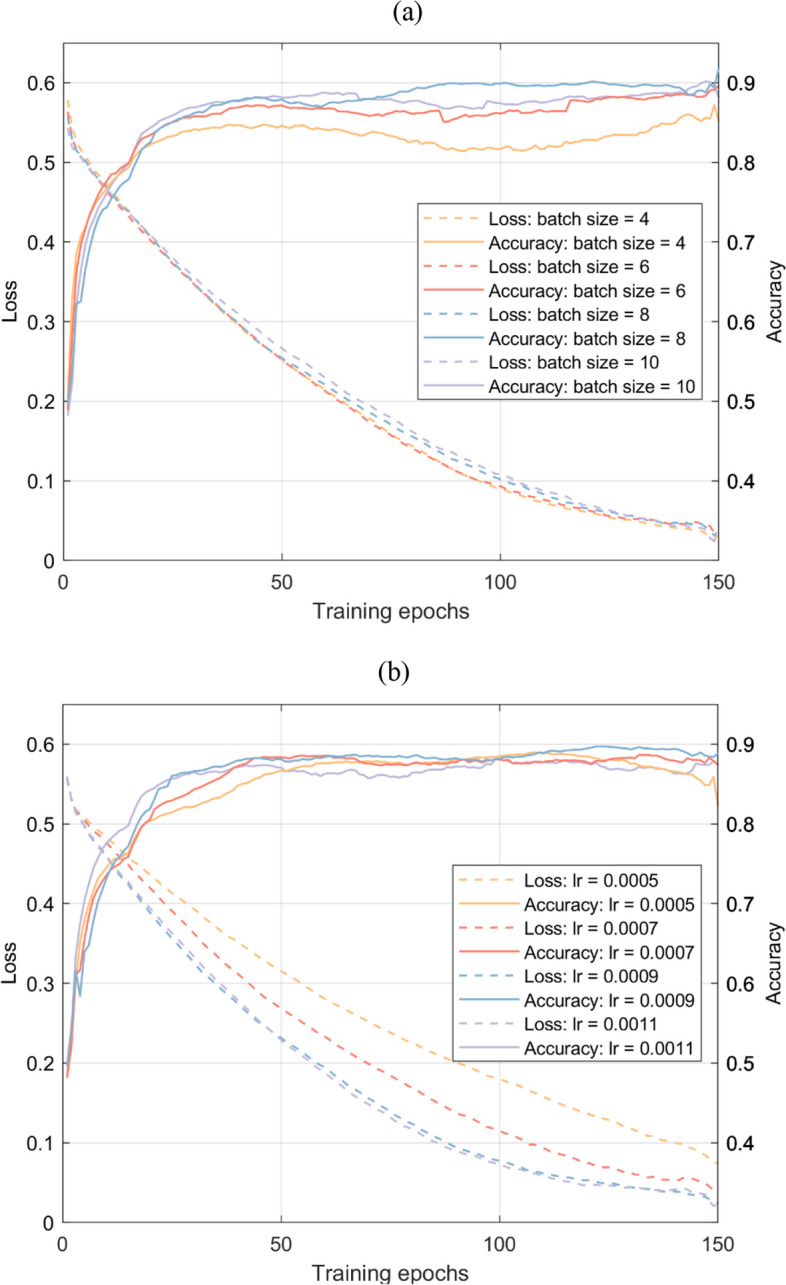
Loss and accuracy curves from hyperparameter tuning (smoothed): **a** learning rate = 0.0009, *L*=6; **b** batch size = 8, *L*=6

According to the optimization results, the final hyperparameters of AE-DenseNet are set as batch size = 8, learning rate = 0.0009, and layer number *L* = 6. To clarify the difference using different ${\varvec{\lambda}}$, Fig. 6 presents the training loss and validation accuracy curves for various hyperparameter combinations. As seen in the figures, changes in batch size have minimal impact on the loss curves but significantly affect accuracy. Smaller batch sizes can hinder the model from learning common features in the signals, while larger batch sizes may cause the model to focus more on noise features. Conversely, an increase in the learning rate speeds up convergence to a lower loss, but beyond a certain point, it can decrease model accuracy.

### Classification Results

With the best hyperparameter combination, the proposed AE-DenseNet was trained with waveform data and applied to damage severity prediction and to identify the potential location of the damage. The location of the damage is identified with respect to the sensor location on the monitored beam by using the statement "damage close or far". For the two tasks, Table 3 tabulates their confusion matrices and corresponding precision and recall rates. The confusion matrix provides a comprehensive summary of the model performance, including the true positives (TPs), true negatives (TNs), false positives (FPs), and false negatives (FNs), which are used to calculate precision and recall rates. The numbers in Table 3 listed in items of "Predicted labels" and "True labels" are the number of samples and represent the confusion matrix. Specifically, for the class "Minor," there are 59 TPs, 6 FPs, and 3 FNs. Then, the precision and recall can be calculated using Eqs. (1) and (2). For the prediction of damage severity, the precision rates for the classes "Minor" and "Severe" are 95.2% and 91.8%, respectively, while the recall rates for them are 90.8% and 95.7%. Regarding the classification of damage locations, the precision rates for the classes "Damage Close" and "Damage Far" are 92.9% and 91.9%, respectively, while the recall rates for them are 96.8% and 82.9%.

**Table 3:** Confusion Matrix, Precision, and Recall rates of the classification results

Damage severity classification				Damage location classification			
Predicted labels	Minor	Severe	Recall	Predicted labels	Damage Close	Damage Far	Recall
True labels				True labels			
Minor	59	6	0.908	Damage Close	91	3	0.968
Severe	3	67	0.957	Damage Far	7	34	0.829
Precision	0.952	0.918	--	Precision	0.929	0.919	--

It should be noted that, in general, closer and more severe damages tend to have higher recognizability (recall) and identification precision. From the above results, close and severe damage cases show a higher recall rate; however, the precision rate of "Severe" damage presents even lower precision. Two reasons likely explain it:

1. The training data used in this study included instances with varying damage severity and different damage locations. However, for some features of ultrasonic waveform signals, the "degree of damage" and "location of damage" can be contradictory. For example, severe damage far from the sensor may exhibit similar amplitude characteristics as minor damage closer to the sensor.
2. The AE signals captured by the large-scale concrete beam during testing are highly complex, and the limited training set sample size prevents the model from reaching its full potential in performance.

Therefore, in the application of AE-based damage detection, it is recommended to predict the damage severity and the damage location separately while increasing the sample size for model training as much as possible.

## Experimental study using a wooden beam

### Experimental setup

**Fig. 7:**
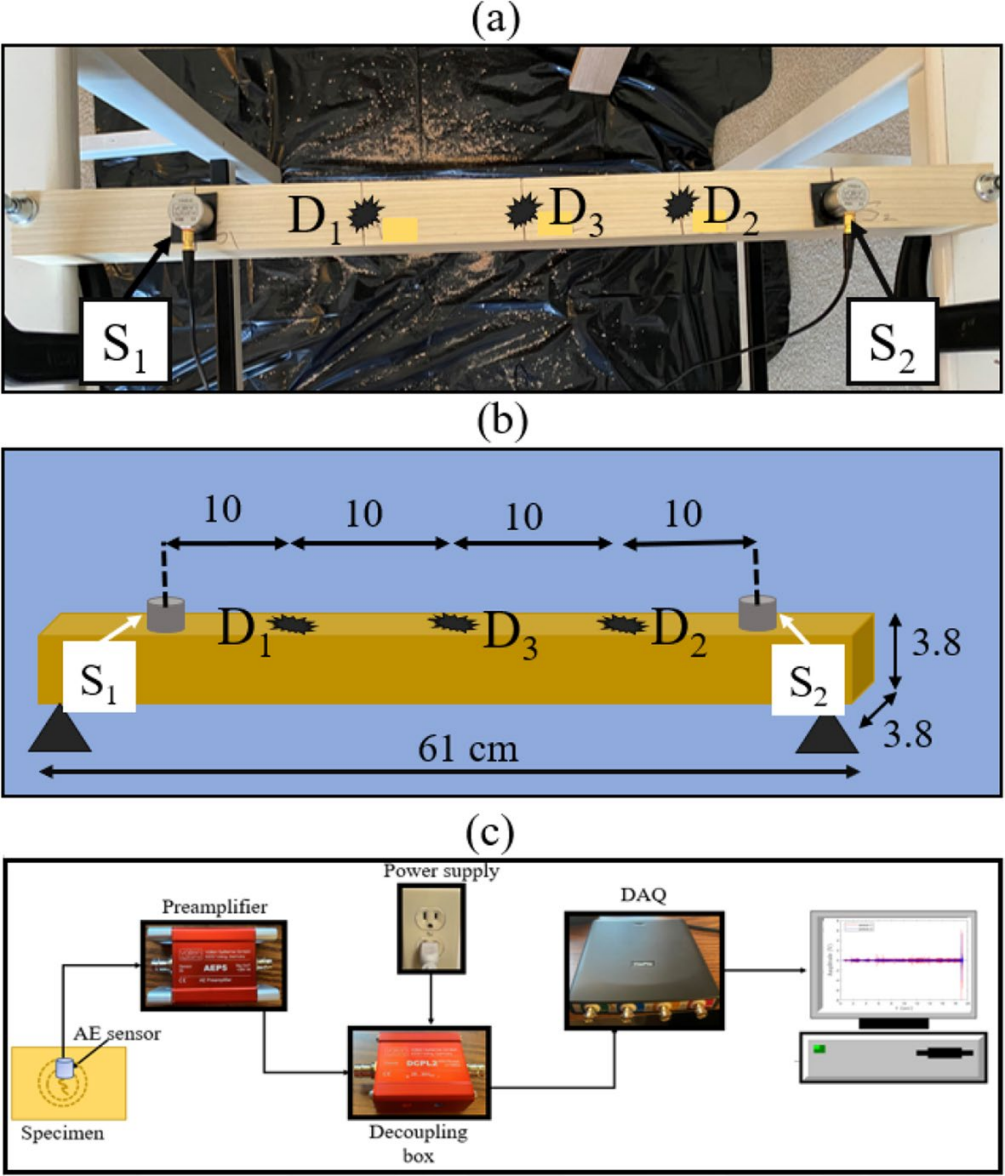
Experimental setup of the specimen **a** actual, **b** the schematic, and **c** the AE monitoring system

In order to evaluate the performance of the proposed method, a wooden beam is considered in the experimental study. The beam measures 61 cm in length and 3.8 cm in width and thickness. Two AE sensors labeled S_1_ and S_2_ are placed on the beam to acquire AE data, as shown in Fig. 7(a). The beam undergoes damage at three distinct locations: D_1_: damage near S_1_; D_2_, damage near S_2_; and D_3_, damage at the center of the beam, as illustrated in Fig. 7(a and b). Damage is induced by simulating a hole using a drilling machine at these locations, while AE sensors collect AE data. The AE monitoring system used to collect AE signals from the wooden beam in this experimental study is shown in Fig. 7(c). It contains two sensors, preamplifiers, decoupling boxes, a data acquisition (DAQ) system, and a computer. The sensors have characteristic frequency bands ranging from 20-450 kHz, where they are connected to preamplifiers with a gain ranging from 34 to 40 dB, coupled with plug-in bandpass filters set between 2.5-2400 kHz. This configuration serves to amplify the AE signal effectively. Also, decoupling boxes are used to connect the preamplifiers with the DAQ system at one end to collect the AE signals and attach them with a direct current supply at the other end to power the AE sensors. The DAQ is connected to a computer to transfer the measured AE signals. With a sampling rate of 200 Msamples/s, the DAQ is suitable for capturing high-frequency AE data collected from the wooden structures. It is important to note that the sensors used in this study operate at a sampling frequency of 20 kHz.

### Data preparation and model training

**Fig. 8:**
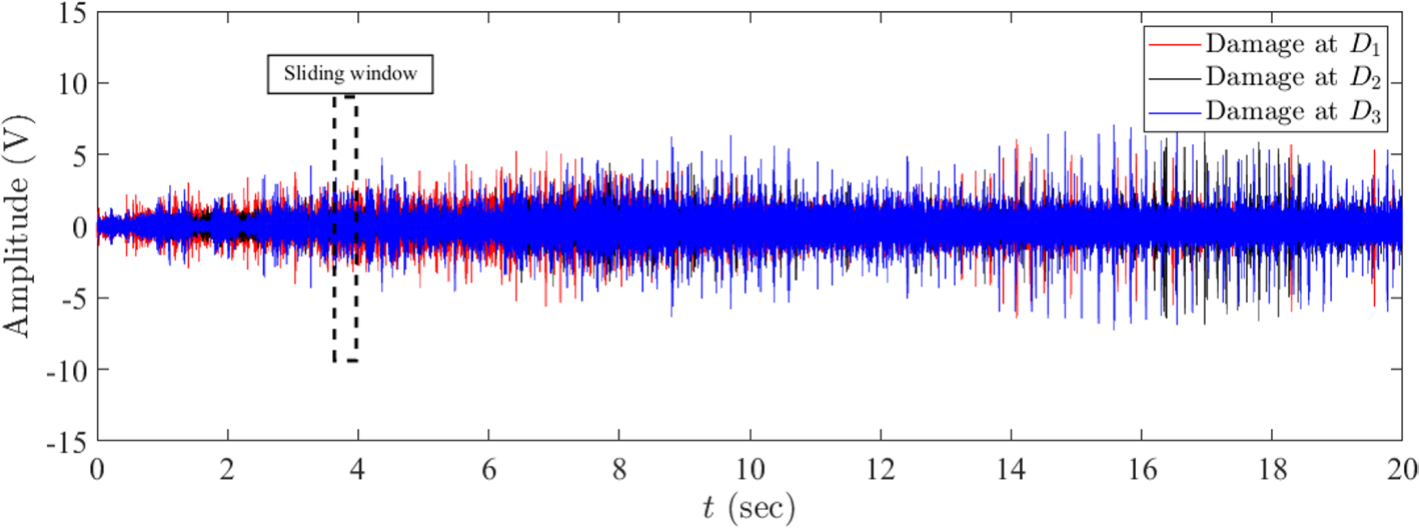
Time history of the measured AE signals collected from the wooden beam using sensor S_1_ for damage at locations D_1_, D_2_, and D_3_, respectively

AE signals collected from sensor S_1_ placed on the wooden beam subjected to active damage are utilized to validate the performance of the proposed method as a damage classification tool. Fig. 8 illustrates the time history of AE signals measured from a wooden beam using S_1_ while damage is applied at locations D_1_, D_2,_ and D_3,_ as discussed in section "Experimental setup". It can be observed that the amplitude of AE signals collected from the beam when the beam is subjected to damage at D_1_ is higher than the amplitude of AE signals when the beam is damaged at locations D_2_ and D_3_. However, It is challenging to differentiate between the amplitude of AE signals collected from the beam due to the damages at locations D_2_ and D_3._ This motivates us to employ the proposed AE-DenseNet method to classify the location of these damages using the time response of AE signals. In order to generate training, validation, and testing data, the data collected during the test is segmented using a sliding window method. The black rectangle shown in Fig. 8 represents the width of the sliding window to generate the segments that are used as input into the AE-DenseNet model.

1000 segments of AE signal for each damage location with 3000 segments in total are generated and then divided by a ratio of 7:2:1 to train, validate, and test the AE-DenseNet for damage identification. The parameter settings discussed in section "Model Training and hyper-parameter Optimization" are used in this section to simplify the training process. The expected location of damages (e.g., D_1_, D_2_, D_3_) on the beam is identified by referring to the location of sensor S_1,_ where AE signals due to the damage at location D_1_ are labelled as "Damage Close" since the actual damage is applied at a distance 10 cm from S_1_, damage at D_2_ are labelled as "Damage Far" since the damage is applied at a distance 30 cm from S_1_, and damage at D_3_ are labelled as "Damage Centred" since the damage is applied at the center of the beam with a distance 20 cm from S_1_, respectively.

### Classification results

The confusion matrix and metrics of the classification results are presented in Table 4. It can be seen that the proposed method can classify the characteristics of AE waveforms collected from one sensor due to damage at different locations on the beam. It can be seen that the proposed AE-DenseNet has the capability to identify the expected damage locations using the time series response of AE signals collected from a single AE sensor (S_1_) with a high accuracy range between 98-100%. The results show the high performance and accuracy of the proposed approach to classify and identify the potential location of damage using the time series response of AE signals, making it a suitable and robust damage identification tool for structures.

**Table 4:** Classification results of the expected location of damage on the wooden beam

Predicted labels	Damage Close	Damage Far	Damage Centred	Recall
True labels				
Damage Close	100	0	0	1.0
Damage Far	2	98	0	0.98
Damage Centred	1	0	99	0.99
Precision	0.971	1.0	1.0	--

## Experimental study using a wooden plate

### Experimental setup

**Fig. 9:**
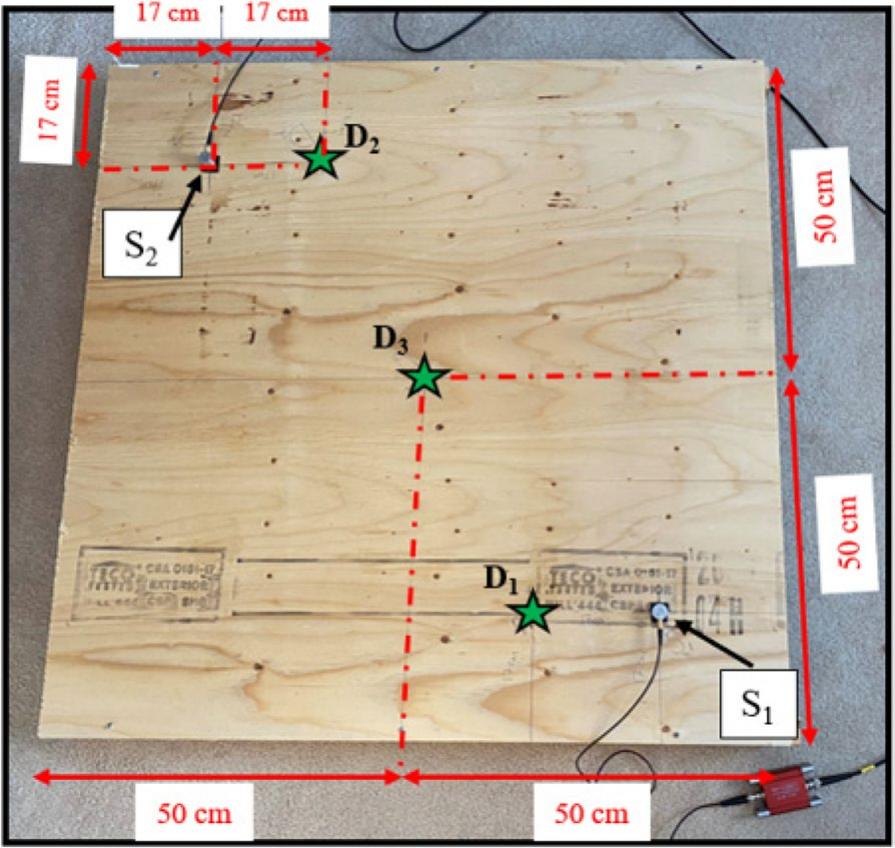
Experimental setup of the specimen

In this section, an experimental test is undertaken to collect AE signals from a wooden plate to verify the performance and efficiency of the proposed approach. The dimensions of the plate are 1 m in length and width, with a thickness of 2 cm. Two sensors (S_1_ and S_2_) are placed at a certain position on the surface of the beam at the same distance from the edge, as shown in Fig. 9. The plate is subjected to damage at three different locations (e.g., location D_1_: damage near S_1_, location D_2_: damage near S_2_, and location D_3_: damage at the center of the plate) as shown in Fig. 9. The plate is damaged by creating a hole using a drilling machine at locations D_1_, D_2_, and D_3_, respectively. The AE signals collected from the wooden plate are acquired using the same AE monitoring system discussed in section "Experimental setup".

### Data preparation and model training

In order to demonstrate the applicability of the proposed approach to different structures further, AE signals induced by damage on a wooden plate are additionally analyzed in this section using the signals collected from one sensor S_1_.

**Fig. 10:**
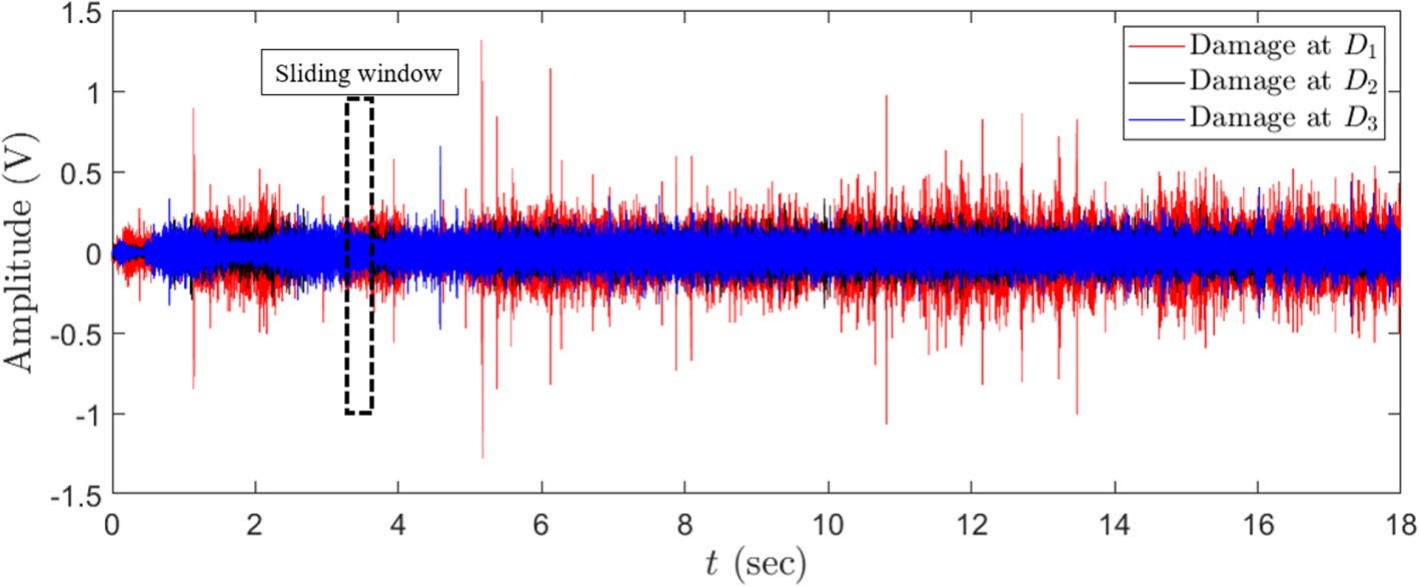
Time history of the measured AE signals collected from the plate using sensor S_1_ for damage at locations D_1_, D_2_, and D_3_, respectively

Fig. 10 presents the time history of AE signals collected from a wooden plate using S_1_ caused by the damages at locations D_1_, D_2,_ and D_3_ (as shown in Section "Experimental setup"). It can be observed that even though the difference between the amplitude of AE signals for damage at D_1_ and D_2_ is visible, the amplitude of AE signals measured from the plate for damage at D_2_ and D_3_ are very similar and are hard to distinguish. Given this, the performance of the proposed AE-DenseNet model in localizing these three types of damage is discussed in this section. A sliding window method is employed to select data segments randomly to obtain sufficient training data. The black rectangle in Fig. 10 represents the width of the sliding window, which corresponds to the input size of the AE-DenseNet. A total of 3000 segments (1000 segments of each damage location) are obtained and divided into the training set, validation set, and testing set by the ratio of 7:2:1. To simplify the training process, the parameter settings from section "Model Training and hyper-parameter Optimization" are maintained.

The identification of potential damage locations involves analyzing AE waveforms collected from the three damage scenarios (D_1_, D_2_, and D_3_), which is considered by referring to the location of sensor S_1_. For example, AE waveforms collected due to the damage at location D_1_ are labelled as "Damage Close" since the actual damage is generated at a distance of 17 cm from S_1_, AE waveforms collected due to the damage at D_2_ are labelled as "Damage Far" since the damage is generated at a distance of 82 cm from S_1_, and AE waveforms collected due to the damage at D_3_ are labelled as "Damage Centred" since the damage is generated at the center of the plate with a distance of 46 cm from S_1_.

### Classification results

Table 5 presents the confusion matrix and metrics of the classification results. It can be seen that the proposed method can differentiate between the characteristics of AE waveforms collected from one sensor due to applying damage at different locations on the plate. It is evident that the proposed AE-DenseNet can identify the expected damage locations by using the time series response of AE signals collected by the same AE sensor (S_1_) with 99-100% accuracy. This demonstrates a significant improvement compared to the authors' previous study [7], in which the identification accuracy of damage at the center of the wooden board, based on Empirical Mode Decomposition, was approximately 86%. Moreover, the identification of the damage location on the wood plate using an images-based CNN model was conducted by Barbosh et al. [9], and the results showed that the accuracy of the model was around 87.5%. The enhanced performance of the proposed approach indicates its superior capability in discerning subtle variations in waveform signals.

**Table 5:** Classification results of damage locations on the wooden plate

Predicted labels	Damage Close	Damage Far	Damage Centred	Recall
True labels				
Damage Close	100	0	0	1.0
Damage Far	0	100	0	1.0
Damage Centred	0	1	99	0.99
Precision	1.0	0.99	1.0	--

## Conclusions

Acoustic emission (AE) method serves as a potent nondestructive technique that has garnered significant attention for its efficacy in detecting and localizing damage in various structural materials, owing to its heightened sensitivity to microcracks. However, the process of damage identification using AE waveforms collected from anisotropic materials such as wood and concrete requires experienced engineers due to its complicated characteristics, which rely on the geometric shape, material uniformity, and effect of background noise. Therefore, damage detection and localization approaches based on the AE parameters and feature selection of the AE signal can be time-consuming and computationally intensive. In this study, the AE signal-based convolutional neural network (CNN) model is proposed to automate the process of damage detection and localization in concrete and wood structures.

The severity of damage and its approximate location are predicted using the automated AE-DenseNet model without considering any manual preprocessing and without involving time-consuming muti-step analysis of the AE data. Experimental studies are conducted using a large-scale concrete beam and wooden beam and plate to validate the performance of the proposed method as a damage severity prediction and localization technique. The extracted results showed the capability of the proposed method for predicting the damage severity using AE waveforms collected from a concrete beam with recall rates between 0.91-0.96. Moreover, the proposed method was able to identify the approximate location of damage in a concrete beam with recall rates ranging between 0.83-0.97 and a wooden beam and plate with accuracy ranges between 98-100%, which makes it a suitable candidate for predicting the damage severity and identifying the expected location of damage in structural elements. The performance of the proposed method was compared to other studies that used feature selection and image-based methods, where the proposed method showed higher accuracy and efficiency. The proposed method has been systematically validated through classification tasks regarding damage location and severity. Based on more detailed classifications, future studies can further identify the coordination of damage location and provide better visualization of the damage in structural elements using AE waveforms.

## Data Availability

The data that support the findings of this study are available from the corresponding author upon request.

## Data availability

No datasets were generated or analysed during the current study.
